# Spatiotemporal Evolution and Spatial Analysis of Ecological Environmental Quality in the Longyangxia to Lijiaxia Basin in China Based on GEE

**DOI:** 10.3390/s24165167

**Published:** 2024-08-10

**Authors:** Zhe Zhou, Huatan Li, Xiasong Hu, Changyi Liu, Jimei Zhao, Guangyan Xing, Jiangtao Fu, Haijing Lu, Haochuan Lei

**Affiliations:** 1 School of Geological Engineering, Qinghai University, Xining 810016, China; zhouzheqhu@sina.com (Z.Z.); liuchangyi1991@sina.com (C.L.); lhcqhdx@163.com (H.L.); 2 School of Water Resources and Civil Engineering, Qinghai University, Xining 810016, China; huatan2008@126.com; 3 College of Agriculture and Animal Husbandry, Qinghai University, Xining 810016, China; zhaojimei@qhu.edu.cn (J.Z.); xingguangyan@qhu.edu.cn (G.X.); luhaijing110@163.com (H.L.); 4 Academy of Agricultural and Forestry Sciences, Qinghai University, Xining 810016, China; fujiangtao865@sina.com

**Keywords:** upper reaches of the Yellow River, Longyangxia to Lijiaxia Basin, Google Earth Engine (GEE), synthesis of GeoDetector and spatial autocorrelation analysis, ecological environment quality (EEQ)

## Abstract

The upper reaches of the Yellow River are critical ecological barriers within the Yellow River Basin (YRB) that are crucial for source conservation. However, environmental challenges in this area, from Longyangxia to Lijiaxia, have emerged in recent years. To assess the ecological environment quality (EEQ) evolution from 1991 to 2021, we utilized remote sensing ecological indices (RSEIs) on the Google Earth Engine (GEE) platform. Spatial autocorrelation and heterogeneity impacting EEQ changes were examined. The results of this study show that the mean value of the RSEIs fluctuated over time (1991: 0.70, 1996: 0.77, 2001: 0.67, 2006: 0.71, 2011: 0.68, 2016: 0.65, and 2021: 0.66) showing an upward, downward, and then upward trend. The mean values of the overall RSEI are all at 0.65 and above. Most regions showed no significant EEQ change during 1991–2021 (68.59%, 59.23%, and 55.78%, respectively). Global Moran’s I values (1991–2021) ranged from 0.627 to 0.412, indicating significant positive correlation between EEQ and spatial clustering, and the LISA clustering map (1991–2021) shows that the area near Longyangxia Reservoir shows a pattern of aggregation, dispersion, and then aggregation again. The factor detection results showed that heat was the most influential factor, and the interaction detection results showed that greenness and heat had a significant effect on regional ecosystem distribution. Our study integrates spatial autocorrelation and spatial heterogeneity and combines them with reality to provide an in-depth discussion and analysis of the Longyangxia to Lijiaxia Basin. These findings offer guidance for ecological governance, vegetation restoration, monitoring, and safeguarding the upper Yellow River’s ecological integrity.

## 1. Introduction

The Yellow River is the second longest river in China, known as “the cradle of Chinese civilization”, with a total length of 5464 km and a total basin area of 752,443 ${\mathrm{km}}^{2}$ [1], providing a large amount of freshwater resources for the coastal population and playing a key role in the development of the regional economy [2]. The Longyangxia to Lijiaxia Basin in the upper reaches of the Yellow River is located between the Loess and Tibetan plateaus, which are the ecological barriers of the Yellow River with intricate tectonic activities, diverse geomorphic features, and fragile natural conditions that are susceptible to the influence of external factors. There are two terrace reservoirs, Longyangxia and Lijiaxia, of which the Longyangxia reservoir is the largest reservoir in the Yellow River Basin (YRB) [3]. Over the past few decades, the combined effects of intense human activity and unfavorable climate change have led to increasingly serious ecological and environmental problems in the upper reaches of the Yellow River, which have seriously affected the ecological security and power supply of the upper reaches of the Yellow River [4,5,6]. Therefore, protecting the fragile ecological environment and making scientific decisions have become key issues plaguing the Longyangxia to Lijiaxia Basin.

Basins play a central role in ecosystems, not only as habitats for human settlements, but also as essential providers of basic ecosystem services, including key functions such as climate regulation, water resource maintenance, biodiversity conservation, and intricate interactions between their ecological elements [7,8,9]. Many scholars have conducted in-depth studies of the importance of basin-level research on ecological services. Gwal et al. estimated and described the Aglar Basin using regionalization techniques for estimating and mapping hydrological ecosystem service (HES) descriptors and temporal analysis of hydrological fluxes using the Soil and Water Assessment Tool (SWAT) [10]. Liu et al. used statistical methods to analyze the spatiotemporal vegetation cover in the YRB and further analyzed the trend of its vegetation cover using Hurst’s index [11]. Jiang et al. constructed an evaluation model covering five aspects—social, economic, resource, ecological, and cultural—and designed four future policy scenarios using a system dynamics (SD) model to simulate changes in the future development of the YRB [12]. Therefore, an in-depth assessment of the ecosystem quality and its spatiotemporal variability at the basin scale is essential for effective ecosystem protection, promoting sustainable regional development, informing targeted conservation efforts, and developing restoration strategies adapted to changing conditions.

Studies have shown that remote sensing technology has become a major tool for quantitative scientific research in the fields of land use, environmental monitoring, and resource management owing to its advantages of speed, wide coverage, and rich information [13,14]. Researchers have widely used remote sensing to dynamically monitor and assess the ecological environment quality (EEQ) of different landscapes such as urban areas, forests, mining areas, basins, and nature reserves [15,16,17]. However, several studies have emphasized that relying on a single indicator to evaluate ecological environments at a regional scale may only reflect specific aspects of the ecosystem. This poses a challenge for a comprehensive and accurate assessment of the overall EEQ. The remote sensing ecological index (RSEI) [18] with multi-indicator integration has been widely used to reveal ecological responses under disturbance conditions and provide a basis for ecological protection management. Spatial analysis, modeling, and prediction functions can be achieved based on the integration of the model with machine learning algorithms such as Gaussian processes (GPs) [19,20]. Xiao et al. [21] used the Muli coal field as the research object, mapped the distribution of the RSEI, and evaluated the EEQ in the mining area. Cai et al. [22] constructed an RSEI, objectively and efficiently assessed the EEQ of the Yangtze River Delta region, and quantitatively analyzed the spatial heterogeneity of the regional EEQ and the interactions between the driving factors using GeoDetector. Yang et al. [23] used the RSEI to assess the EEQ of Kunming and Nanjing and explored the EEQ of the mining area and changes in its landscape pattern. The above studies show that RSEI not only integrates multiple indicators for comprehensive evaluation but also intuitively reflects the temporal and spatial changes of EEQ, with strong reliability and applicability.

Since 2010, the Google Earth Engine (GEE), a free open-access platform designed for research, education, and non-profit activities [24], has replaced the traditional local processing of remote sensing images [25]. GEE has been widely used, particularly in large-scale fields, such as on a global scale [26]. Users can process these data directly on the platform using resource computing. Relevant datasets (e.g., the Landsat series) have already been preprocessed and can be further analyzed without the need for dedicated software [27]. Compared to traditional remote sensing data processing tools, the GEE is more suitable for basin-scale EEQ assessments of the RSEI.

Although existing environmental quality assessment methods can effectively identify regional ecological conditions, they tend to overemphasize numerical results and regional characteristics, making it difficult to accurately capture the dynamic spatial distribution and heterogeneity of ecosystems. Moreover, current studies are often based on static comparisons of two moments, thus ignoring their dynamic trajectories, and are limited to studying the spatial autocorrelation or heterogeneity of environmental quality within a single watershed, failing to combine the two in a comprehensive analysis [28]. To address this gap, our study synthesized spatial autocorrelation and heterogeneity and integrated them with the current state of EEQ changes in the upper reaches of the Yellow River. The objectives of this study were to: (1) integrate remote sensing data based on the GEE platform, leading to efficient construction of the RSEI; (2) identify spatiotemporal variations in EEQ in the Longyangxia to Lijiaxia Basin and the upper reaches of the Yellow River and to reconstruct and analyze time series changes in the EEQ from 1991 to 2021; (3) analyze the spatial differentiation characteristics of EEQ from the Longyangxia to Lijiaxia Basin; (4) explore the individual effects and interactions among the four RSEI factors and their respective impacts on the change in EEQ. Our study comprehensively explored the ecological environment from Longyangxia to the Lijiaxia Basin to provide a scientific foundation for EEQ protection, management, and decision-making.

## 2. Materials and Methods

### 2.1. Study Area

**Figure 1:**
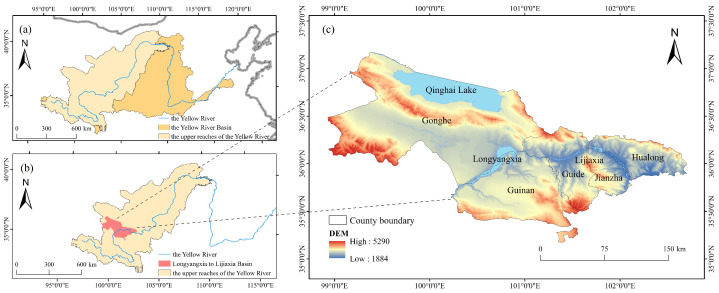
Location of the Longyangxia to Lijiaxia Basin. (**a**) Location of the YRB and the upper reaches of the Yellow River. (**b**) Location of the upper reaches of the Yellow River and the Longyangxia to Lijiaxia Basin. (**c**) Regional map of the Longyangxia to Lijiaxia Basin.

The Longyangxia to Lijiaxia Basin (35°09′–37°12′ N, 99°–102°42′ E) is located in the upper reaches of the Yellow River, in the southeastern part of Qinghai Province, covering the five counties of Gonghe, Guide, Guinan, Jianzha, and Hualong, as shown in Figure 1. In the study area, the altitude ranges from 1884 to 5290 m, the average annual temperature ranges from 1.2 to 9 °C, and the average annual precipitation ranges from 300 to 566.2 mm [29]. The average annual evaporation during 1957–2010 was approximately 1500 mm [30].

### 2.2. Data and Preprocessing

**Figure 2:**
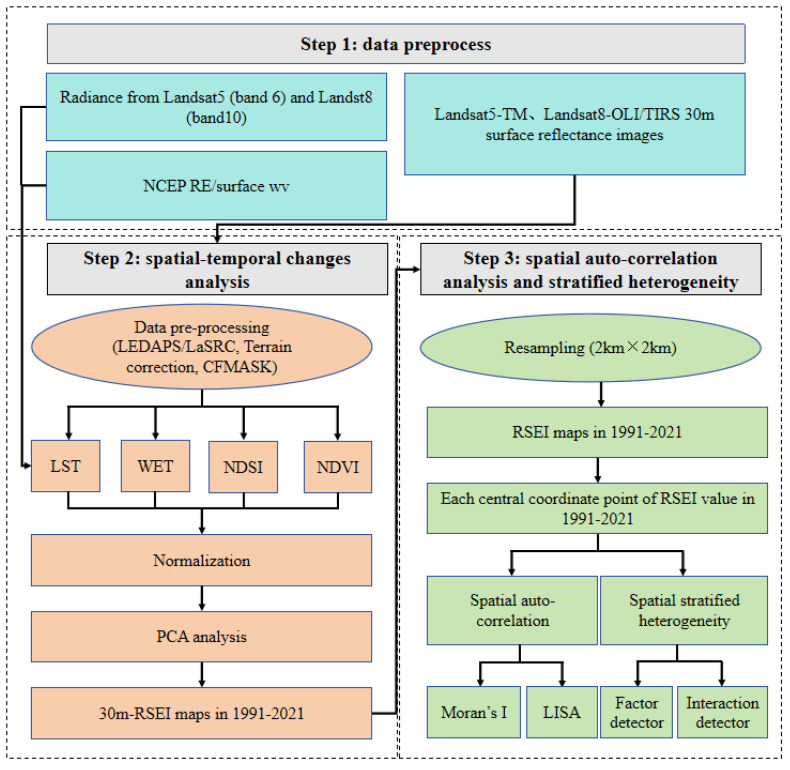
Workflow of data processing.

A detailed workflow was established for this study (Figure 2). First, we produced seven 30 m RSEI maps for 1991, 1996, 2001, 2006, 2011, 2016, and 2021 using Landsat 5 TM and 8 OLI/TIRS images based on the GEE platform. Second, based on the seven RSEI maps from 1991–2021, the spatiotemporal characteristics of EEQ in the Longyangxia to Lijiaxia Basin were analyzed. The spatial correlation of the EEQ was analyzed using Moran’s I (global spatial autocorrelation) and the Local Indicators of Spatial Association (LISA) indices. Finally, the effects of individual factors and their interactions on EEQ were explored using GeoDetector.

### 2.3. Methodology

#### 2.3.1. Establishment of Remote Sensing Ecological Index

Within the array of natural factors signifying the EEQ, greenness, wetness, dryness, and heat have intricate interconnections with human sustenance, establishing them as pivotal indicators within the intuitive human perception of ecological states. Consequently, these parameters are frequently used in EEQ assessment [31,32,33]. The RSEI was formulated by combining the greenness (normalized difference vegetation index; NDVI), wetness (WET), dryness (normalized difference soil index; NDSI), and heat indices (land surface temperature; LST). This comprehensive amalgamation quantitatively represented the EEQ and its alterations. The formula is as follows:

(1) $$\begin{array}{c} RSEI=f(NDVI,WET,NDSI,LST). \end{array}$$

The modified normalized difference water index (MNDWI) was utilized to demarcate the water body region before calculating the RSEI. This step was performed to ensure that WET accurately reflected the wetness characteristics within the study region [6]. The mathematical expressions for these indices are outlined in Table 1.

We used the normalization function in the GEE to account for the different units and scales of the four indicators, resulting in values in the [0,1] interval. This standardization was performed to facilitate comparative analysis. Subsequently, principal component analysis (PCA) was applied to the image data to restrict the four normalized metrics to the [0,1] range [34]. Then, the GEE platform was used for feature analysis to calculate the first principal component (PC1). Developers created libraries within the GEE framework to perform a PCA for subsequent analyses. The formula is as follows:

(2) $$\begin{array}{c} RSEI_{0}=1-PCA\left[f\left(NDVI,WET,NDSI,LST\right)\right], \end{array}$$

where $RSEI_{0}$ denotes the initial ecological index derived using PCA. The RSEI was normalized by restricting the value of $RSEI_{0}$ to a range of [0,1]. The higher the RSEI value, the better the EEQ is. Conversely, the closer the RSEI value is to 0, the poorer the EEQ is [18].

**Table 1:** WET, NDVI, NDSI, LST, and MNDWI formulas and explanations.

Index	Formula	Explanation
WET	WET=c1B1+c2B2+c3B3=+c4B4+c5B5+c6B6	WET is a measure of transformed humidity that is indicated by the Tasseled Cap Transformation (TCT); $B_{1}-B_{6}$ are reflectance values of the blue, green, red, near-infrared, shortwave infrared 1, and shortwave infrared 2 bands, respectively; $c_{1}-c_{6}$ are sensor parameters [35,36].
NDVI	$NDVI=\frac{\rho_{\mathrm{NIR}}-\rho_{R}}{\rho_{\mathrm{NIR}}+\rho_{R}}$	NDVI is a commonly used vegetation index for visualizing plant cover; $\rho_{\mathrm{NIR}}$ and $\rho_{R}$ represent reflectance values of the near-infrared and red bands, respectively [37,38].
NDSI	$NDSI=\frac{SI+IBI}{2}$ $SI=\frac{\left(\rho_{\mathrm{NIR}}+\rho_{R}\right)-\left(\rho_{\mathrm{NIR}}+\rho_{B}\right)}{\rho_{\mathrm{NIR}}+\rho_{R})+\left(\rho_{\mathrm{NIR}}+\rho_{B}\right)}$ $IBI=\frac{\frac{2\rho_{\mathrm{SWIR}1}}{\rho_{\mathrm{SWIR}1}+\rho_{\mathrm{NIR}}}-\frac{\rho_{\mathrm{NIR}}}{\rho_{\mathrm{NIR}}+\rho_{R}}-\frac{\rho_{G}}{\rho_{G}+\rho_{\mathrm{SWIR}1}}}{\frac{2\rho_{\mathrm{SWIR}1}}{\rho_{\mathrm{SWIR}1}+\rho_{\mathrm{NIR}}}+\frac{\rho_{\mathrm{NIR}}}{\rho_{\mathrm{NIR}}+\rho_{R}}+\frac{\rho_{G}}{\rho_{G}+\rho_{\mathrm{SWIR}1}}}$	NDSI is calculated by averaging the Index-based Built-up Index (IBI) and Soil Index (SI); $\rho_{\mathrm{SWIR}1}$, $\rho_{\mathrm{NIR}}$, $\rho_{R}$, and $\rho_{B}$ are reflectance values of the shortwave infrared 1, near-infrared, green, red, and blue bands, respectively [39,40].
LST	$T=\frac{K_{2}}{\ln\left(\frac{K_{1}}{L_{6}}+1\right)}$ $LST=\frac{T}{1+\ln\left(\varepsilon \left(\frac{\lambda T}{\rho }\right)\right)}-273$	LST is the surface temperature obtained from the atmospheric correction method and inverted according to the Landsat manual and the corresponding parameters; *T* is the temperature at the sensor and ρ is $1.438\times 10^{-2}$ m·K; ε is the surface specific emissivity; $K_{1}$ and $K_{2}$ are the predefined calibration parameters at the time of the satellite launch, respectively; λ is the central wavelength of the thermal infrared band; and $L_{6}$ is the reflectance of the thermal infrared radiation of the remotely sensed image after calibration [32,41].
MNDWI	$MNDWI=\frac{\rho_{G}-\rho_{\mathrm{NIR}}}{\rho_{G}+\rho_{\mathrm{NIR}}}$	$\rho_{G}$ and $\rho_{\mathrm{NIR}}$ are reflectance values of the green and mid-infrared bands, respectively [42].

#### 2.3.2. Spatial Auto-Correlation Analysis

Spatial autocorrelation constitutes a pivotal metric for evaluating the extent to which the attribute values of an entity demonstrate a notable correlation with those of proximate spatial entities. It identifies correlation patterns among attribute feature values across spatial reference units and their neighboring counterparts [43,44]. In this study, Moran’s I and LISA were used to analyze spatial autocorrelation.

Moran’s I index can be used as a coefficient to assess the correlation of attribute values between cells that are spatial neighbors and can be calculated using GlobalMoran’s. The strength of spatial autocorrelation is reflected by the proximity of the absolute value of Moran’s I to 1, which is calculated as follows [45]:

(3) $$\begin{array}{c} \mathrm{GlobalMoran}’s\;I=\frac{m\sum_{i=1}^{m}\sum_{j=1}^{m}\omega_{ij}\left(D_{i}-\bar{D}\right)\left(D_{j}-\bar{D}\right)}{\sum_{i=1}^{m}\sum_{j=1}^{m}\omega_{ij}{\left(D_{i}-\bar{D}\right)}^{2}}, \end{array}$$

where *m* denotes the aggregate quantity of elements, $D_{i}$ signifies the EEQ value at the *i*-th geographical location, and $\bar{D}$ represents the mean EEQ across all elements within the investigated area. Spatial interactions are determined through the utilization of $\omega_{\mathrm{ij}}$ as the spatial weight. Moran’s I, a metric bounded within the interval [−1,1], serves as a quantitative indicator of spatial correlation. A proximity to the value of 1 in Moran’s I denotes an intensified positive spatial correlation in the EEQ, whereas an approximation of −1 indicates a heightened negative spatial correlation. A value of zero signifies the absence of a discernible spatial autocorrelation in the EEQ [46].

The LISA index reflects local spatial autocorrelation and can be used for further spatial autocorrelation analysis based on Moran’s I index. It analyzes the value of the Moran’s I index on a spatial unit, reflecting the local spatial correlation. LocalMoran’s I can be used to calculate this. It identifies sites that may hide local spatial autocorrelations in the absence of global ones. Conversely, in cases where global spatial autocorrelation was evident, the analysis was extended to the presence of spatial heterogeneity. The formula is as follows:

(4) $$\begin{array}{c} LocalMoran^{\prime }s\;I=\frac{\left(D_{i}-\bar{D}\right)\times \sum_{j=1}^{m}\omega_{ij}\left(D_{j}-\bar{D}\right)}{\sum_{i=1}^{m}{\left(D_{i}-D\right)}^{2}}, \end{array}$$

where *LocalMoran’s I* represents the LocalMoran’s I index, and its calculation parameters are the same as those of GlobalMoran’s I.

The LISA clustering diagram delineates five specific types of local spatial clustering: high–high (H–H), low–low (L–L), low–high (L–H), high–low (H–L), and not significant. H–H clustering indicates that both the focal and surrounding areas have high EEQ. In instances of L–L clustering, both the EEQ of the focal area and its neighboring regions are low. L–H clustering indicates low EEQ in the focal area, juxtaposed with high quality in adjacent areas. Conversely, H–L clustering implies high EEQ in the focal area, in contrast to the low quality in its surrounding areas [34].

Spatial correlation analysis was performed to identify variables with spatial correlations and to evaluate their strength. To ensure the precision of the data at an appropriate scale and to conduct accurate quantitative assessments, this study employed a 2 km × 2 km grid based on the landscape pattern and ecosystem characteristics of the study area for image resampling [34]. We systematically collected 6643 sample points from each RSEI image in 1991, 1996, 2001, 2006, 2011, 2016, and 2021 and used them to analyze the spatial autocorrelation of the RSEI in the upper reaches of the YRB from Longyangxia to Lijiaxia.

#### 2.3.3. GeoDetector

GeoDetector is a statistical method that can determine the spatial heterogeneity of geographic phenomena in a region, explain potential factors, and effectively identify the consistency of the spatial distribution of variables and causality [47,48]. We used GeoDetector to analyze spatial dissimilarities, detect explanatory factors, and analyze the interactions between RSEI and NDVI, WET, NDBI, and LST in the study area. The formulas for the factor detector are as follows:

(5) $$\begin{array}{c} q=1-\frac{\sum_{h=1}^{L}N_{h}\sigma_{h}^{2}}{N\sigma^{2}}=1-\frac{SSW}{SST}, \end{array}$$

(6) $$\begin{array}{c} SSW=\sum_{h=1}^{L}N_{h}\sigma_{h}^{2}, \end{array}$$

(7) $$\begin{array}{c} SST=N\sigma^{2}, \end{array}$$

where *q* represents a statistical measure of the explanatory power of the independent variables with values in the range [0,1]; *h* represents the classification interval of each factor; *L* is the total number of layers in the classification; $N_{h}$ and *N* are the number of units in layer *h* and the total number of units in the entire area, respectively; $\sigma_{h}^{2}$ and $\sigma_{2}$ are the variances of RSEI values in layer *h* and the entire area, respectively; and *SSW* and *SST* represent the cumulative variances within individual layers and the aggregate variance across the entire spatial domain.

Interaction detectors are effective at identifying the interactions between different risk factors, and the collective impact of two factors can be assessed to determine whether the explanatory power of the dependent variable is enhanced or diminished by detecting whether the effects of these factors on the dependent variable are interdependent. Methods for assessing interactions require calculating the explanatory validity of individual factors and then calculating the explanatory power resulting from the interaction between two factors such that their respective explanatory powers can be analyzed and compared.

We used four indices—NDVI (X1), WET (X2), NDBI (X3), and LST (X4)—obtained at the same temporal scales from 1991 to 2021 as the independent variables. The RSEI values were designated as dependent variables, and the stratification method was applied. The independent variable values were transformed from numerical values into categorical variables through the application of a stratification method and were then categorized into five different categories using the natural break method. A grid was constructed to obtain 6643 sample points. In addition, the values of the dependent variable RSEI were juxtaposed with the values of the four independent variables for each sample point and analyzed using GeoDetector to obtain the explanatory power of the respective variables on the spatial distribution within the region (*q* value), where the *q* value can effectively reflect the influence of factors on the spatial distribution. We used the Excel GeoDetector software for the analysis, which can be accessed from the website (http://www.geodetector.cn/, accessed on 1 January 2023) [47].

## 3. Results

### 3.1. Principal Component Analysis Results

As shown in Table 2, the contribution of PC1 to the seven historical images from 1991–2021 was greater than 95%, with PC1 being 96.61%, 97.75%, 97.32%, 97.58%, 97.50%, 97.30%, and 96.23% for 1991, 1996, 2001, 2006, 2011, 2016, and 2021, respectively, indicating that PC1 concentrated most of the characteristics of the four component indices. In PC1, the eigenvalues of NDVI and WET are positive, indicating that they play a positive role in RSEI, while the eigenvalues of NDSI and LST are negative, indicating that they have a negative impact on RSEI, which is in line with the actual situation [8].

### 3.2. Overall Evaluation of EEQ in the Longyangxia to Lijiaxia Basin

**Figure 3:**
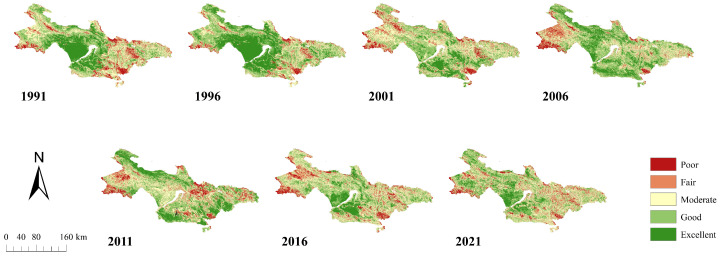
Spatial distribution of EEQ levels from the Longyangxia to Lijiaxia Basin from 1991 to 2021.

Figure 3 depicts the spatiotemporal variability attributes of the EEQ within the Longyangxia to Lijiaxia Basin. As illustrated, the overall EEQ in the region remained consistently favorable in 1991. Regions with poor and moderate ecological grades are predominantly situated in the northern part of Jianzha County, the southwestern part of Gonghe County, and the southern part of Guide County, which are characterized by lower elevation and frequent human activities. Conversely, areas with excellent ecological grades are localized within the western expanse of the study area, where the elevation is higher, forest coverage is substantial, and urbanization is limited. Further basin analysis revealed that the upper reaches primarily exhibited poor and fair EEQ, the middle reaches showed excellent and good quality, and the lower reaches were mainly characterized by fair and good quality.

**Figure 4:**
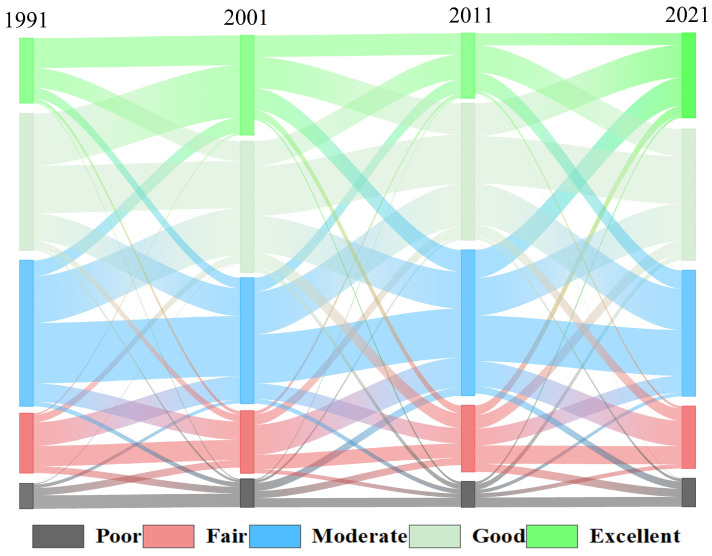
Sankey diagram of EEQ levels for the Longyangxia to Lijiaxia Basin from 1991 to 2021.

Subsequent analysis revealed a discernible temporal trend in the mean RSEI values in the region, indicating it fluctuates from decreasing–increasing–decreasing in 1991–2021. Noteworthy improvements were observed between 2001 and 2006. By employing a series of seven RSEI maps at 5-year intervals spanning 1991 to 2021, comprehensive calculations were undertaken to determine the extent and proportionality of each ecological classification, encompassing the categories of poor, fair, moderate, good, and excellent. Figure 4 illustrates the proportional changes for each ecological grade. From 1991 to 2001, there was a reduction in the overall proportion of ecological grades categorized as poor, fair, and moderate, juxtaposed with a concurrent increase in the proportion of grades classified as good or excellent. Between 2001 and 2011, the proportion of poor or excellent ecological ratings decreased, whereas the proportion of moderate to good ratings increased. However, from 2011 to 2021, the ecological ratings of good and excellent increased again. In summary, the EEQ within the basin spanning from Longyangxia to Lijiaxia exhibited a progression characterized by amelioration from 1991 to 2001, followed by regression from 2001 to 2011, and a resurgence from 2011 to 2021.

Additionally, we computed the area of each RSEI grade and the sum of the proportions for the poor, fair, and moderate (PFM%) and good and excellent (GE%) ecological levels, as presented in Table 3. The PFM% for the years 1991, 1996, 2001, 2006, 2011, 2016, and 2021 was 48.73%, 34.41%, 53.38%, 43.19%, 50.09%, 56.50%, and 54.55%, respectively. This indicates a changing trend consisting of an initial increase and subsequent decrease in PFM% and an initial decrease and subsequent increase in GE%.

### 3.3. Analysis of Spatiotemporal Differences in EEQ

Table 4 shows the performance and spatiotemporal variations in the RSEI across the Longyangxia to Lijiaxia Basin over three distinct periods: 1991–2001, 2001–2011, and 2011–2021. This study systematically assessed variances in RSEI grades by categorizing the outcomes into five distinct levels: improvement obvious (IO), improvement slight (IS), invariability (IN), deterioration slight (DS), and deterioration obvious (DO). The largest proportion of these levels was IN with 68.59%, 59.23%, and 55.78%, respectively. Thus, a variation in the EEQ in the study area was characterized by more than half of the area remaining stable.

In addition, the IO and DO values for ecological quality in the study area were less than 3% between 1991 and 2021. This indicates that a few zones exhibited significant changes during this period. Notably, the ratio of IS to DS constantly changed, increasing from 12.11% in 1991–2001 to 20.34% in 2001–2011, and then decreasing to 19.86% in 2011–2021. Furthermore, the percentage of DS changed from 18.22% in 1991–2001, decreased to 17.77% in 2001–2011 and increased to 21.71% in 2011–2021. These results show an overall significant trend of increasing the proportion of regions with EEQ level changes, with DS and IS dominating the levels of change.

### 3.4. Spatial Autocorrelation Results and Analysis

**Figure 5:**
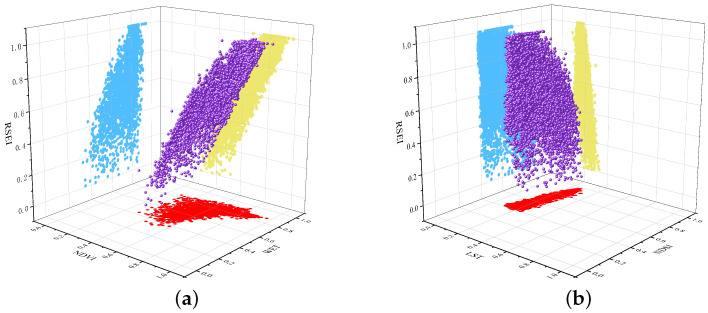
Three-dimensional scatter plots of indicators. (**a**) NDVI and WET show a positive correlation. Red represents the relationship between NDVI and WET, blue represents the relationship between RSEI and WET, and yellow represents the relationship between RSEI and NDVI. (**b**) LSI and NDSI show a negative correlation. Red represents the relationship between LST and NDSI, blue represents the relationship between RSEI and NDSI, and yellow represents the relationship between RSEI and LST.

Building on this foundation, the relationship between each indicator and RSEI, along with the examination of positive (NDVI and WET) and negative indicators (LST and NDSI), were scrutinized. The RSEI samples were projected onto a three-dimensional coordinate axis. As depicted in Figure 5a, elevated NDVI and WET values aligned with higher RSEI values, highlighting a positive correlation between greenness, wetness, and EEQ. Conversely, Figure 5b illustrates that increased LST and NDSI values corresponded to lower RSEI values, indicating a negative correlation between dryness, heat, and EEQ.

**Figure 6:**
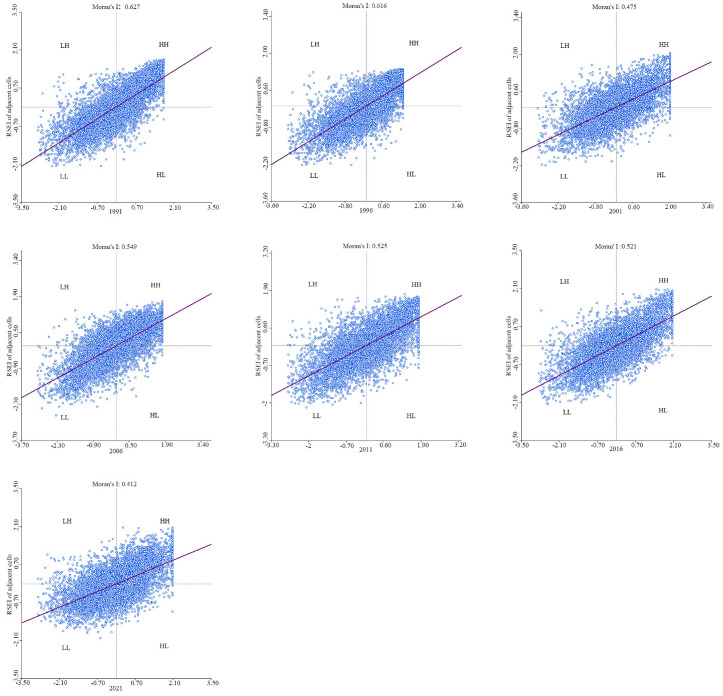
Moran scatter plots of the RSEI in the Longyangxia to Lijiaxia Basin in 1991, 1996, 2001, 2006, 2011, 2016, and 2021.

This analysis employed both the Moran’s I index and LISA. The scatter plot illustrating Moran’s I for the RSEI, presented in Figure 6, delineates a positive spatial correlation in the EEQ within the study area. The scatter points were primarily concentrated within the first and third quadrants for each year. Moreover, the Moran’s I index values for 1991, 1996, 2001, 2006, 2011, 2016, and 2021 were determined to be 0.627, 0.616, 0.475, 0.549, 0.525, 0.521, and 0.412, respectively. These values indicate a notable tendency towards clustering rather than a stochastic distribution of the EEQ throughout the spatial domain. In examining the temporal dimension, Moran’s I exhibited a declining trajectory from 1991 to 2001, subsequently displayed an ascending pattern from 2001 to 2006, and eventually reverted to a diminishing trend from 2006 to 2021. This temporal evolution is consistent with the pattern observed in the EEQ.

**Figure 7:**
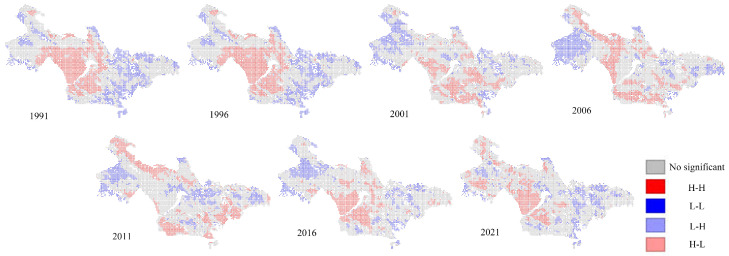
LISA cluster map of the RSEI in the Longyangxia to Lijiaxia Basin in 1991, 1996, 2001, 2006, 2011, 2016, and 2021.

LISA clustering maps were used to identify local spatial correlation patterns and perform a local autocorrelation analysis to comprehensively identify the spatiotemporal distribution characteristics of EEQ in the study area. As shown in the LISA clustering diagram in Figure 7, the areas categorized as “no significant” are located primarily in the middle and high elevations. The H–H clustering areas were prominent along the Longyangxia coast in 1991 and 1996. However, from 2001 to 2021, the majority of the Longyangxia coast transitioned into L–L states with no significant clustering, indicating a notable decline followed by an increase in EEQ. Additionally, originating from the vicinity of the Longyangxia Reservoir, there was a pattern of initial concentration, followed by dispersion and subsequent concentration. From 1991 to 2021, the H–H aggregation area first decreased and then increased, which was consistent with the observed regional changes in EEQ, whereas areas with L–L aggregation characteristics were mainly distributed along the coast of the basin and in the western region, with high population density and human activity. This was consistent with the spatial distribution of lower EEQ in areas with higher human activity.

### 3.5. Quantitative Analysis of Impact Factors in EEQ

#### 3.5.1. Analysis of Discrepancies in Singular Factors

The *p* values for the four factors influencing the RSEI in the region were all zero, as indicated in Table 5, signifying the significant impact of all four factors on the RSEI. Additionally, factor detection analysis revealed a descending order of explanatory power from 1991 to 2021: LST, WET, NDSI, and NDVI. The *q* value of LST was notably large, signifying a substantial difference compared with the other factors and highlighting the pronounced spatial distribution impact of soil desiccation in the region. NDVI, WET, NDSI, and LST exhibited decreasing trends over time. Even when the LST registered a decrease below 0.5, its impact on the EEQ in the region remained substantial.

#### 3.5.2. Analysis of Differences in Factor Interactions

**Figure 8:**
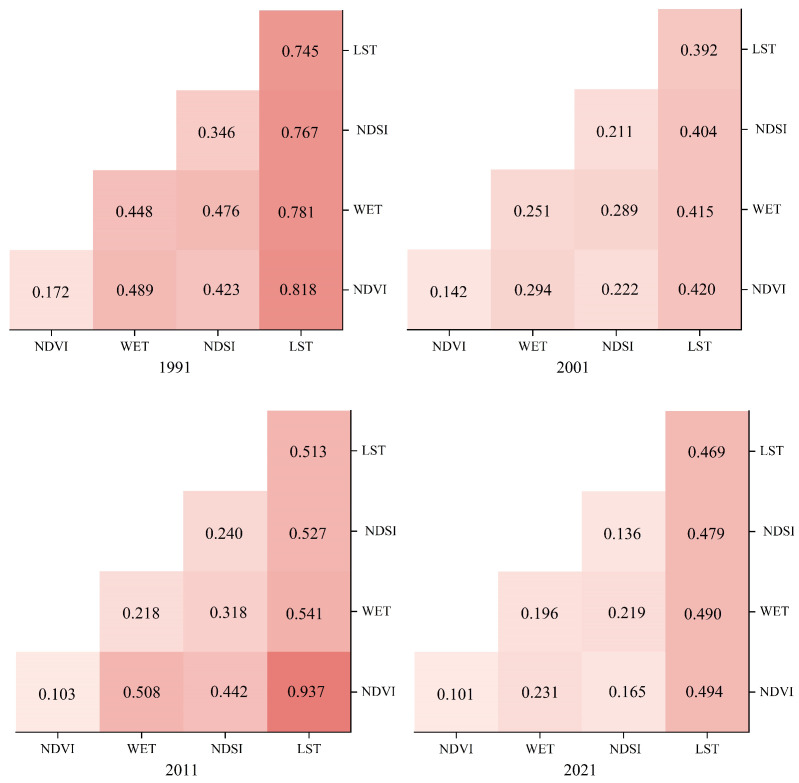
Detecting the results of the interaction of four indicators in 1991, 2001, 2011, and 2021.

As illustrated in Figure 8, the region exhibits a consistent linear enhancement trend in the four indicators from 1991 to 2021, which is influenced by the interaction between the two factors. The impact of the interaction between the two factors on the EEQ was more pronounced than that of the individual factors. Integrated analysis revealed that, from 1991 to 2021, the interaction between NDVI and LST had the most substantial impact on regional EEQ. This is evident from the *q* values for 1991, 2001, 2011, and 2021, which were 0.818, 0.420, 0.937, and 0.494, respectively. Notably, the *q* values of the interaction between NDVI and LST in 1991 and 2011 were 0.818 and 0.937, respectively, signifying a significant mutual influence of greenness and heat on the spatial distribution of the ecological environment in the study area. The results show that the interaction of greenness and heat indicators had the largest effect on EEQ from 1991 to 2021.

## 4. Discussion

### 4.1. Influence of Natural Conditions and Human Activities on the Ecological Environment

The changes in EEQ before 2001 were mainly influenced by both natural factors and human activities, with the heat factor having the most significant impact due to frequent human activities in recent years and the melting of glaciers on the Tibetan Plateau. These led to the greenhouse effect, resulting in higher temperatures, thereby impacting on EEQ [49,50]. Meanwhile, due to the intensification of human activities in the last 30 years, which has greatly increased the built-up area of the upper reaches of the Yellow River, and due to irrational land use, which has increased the area of bare land, all these factors affect the dryness indicator, thus affecting the change in EEQ [51]. Furthermore, as the upper reaches of the Yellow River are in arid and semi-arid areas, the vegetation itself is sparse and susceptible; not only do human activities encroach on a large area of vegetation resources, but the process of urbanization in some areas produces the consumption of resources, all of which have an important impact on the vegetation cover [52,53,54,55], and precipitation is also an important factor affecting the change in EEQ. Human water use in the upper reaches of the Yellow River can lead to a decrease in surface water reserves, while the over-exploitation of groundwater can also lead to a decrease in surface runoff, all of which will either indirectly or directly affect the precipitation pattern in the upper reaches of the Yellow River, thus affecting the change in EEQ [56,57].

The change in EEQ after 2001 was influenced by human activities [58]. During these years, the government carried out land use planning and effectively controlled the process of urbanization. At the same time, it also carried out the policy of rational and environmental water resource utilization, such as the “reclaimed water recycling project” [59]. In terms of ecological restoration, the government has implemented the policy of “Returning Farmland to Forests or Grasslands” [60,61], which realizes the restoration of vegetation and woodlands, an initiative that not only prevents land desertification but also reduces soil erosion and other phenomena that play an important role in the restoration of EEQ [62]. In addition, government initiatives to rationalize the planning of groundwater extraction can also increase surface runoff, thereby affecting the pattern and distribution of precipitation.

From a regional perspective, the EEQ spatial concentration pattern of the Longyangxia to Lijiaxia Basin changes more frequently, and it is recommended that treatment and restoration start from the coast of the basin. From the results of the factor test, the heat factor is the most important factor, and it is recommended to rationally allocate resources, carry out conservation farming, build an agroforestry composite system, and implement other initiatives in order to reduce the greenhouse effect. On this basis, it is also necessary to rationally adjust the land use pattern and urbanization process of the cities along the Longyangxia to Lijiaxia Basin and reduce the area of bare land so as to achieve the coordination between human activities and the natural environment. At the same time, the protection of water resources in the upper reaches of the Yellow River should be emphasized to adapt to its special natural environment, and drought-resistant trees and shrubs can be introduced for planting as a means of vegetation protection and restoration.

### 4.2. Advantages and Constraints

We constructed the RSEI based on the GEE platform to assess the evolutionary characteristics of the EEQ in the upper YRB from Longyangxia to Lijiaxia from 1991–2021. Our results showed that the regional green and humidity indices of the EEQ played a positive role (Figure 5a), whereas the dryness and heat indices played negative roles (Figure 5b) [63]. These results are consistent with RSEI patterns observed in forests [64], mining areas [65], cities [66], and other environments [28]. Our study confirms that the RSEI model can be widely applied to the assessment of EEQ at the basin scale and that the addition of the GEE platform can significantly improve efficiency and save computational resources. In addition, by analyzing the absolute values of NDVI, WET, NDSI, and LST from 1991 to 2021 (Table 5), the absolute value of the dryness index was found to be the largest. Therefore, temperature may be the main factor affecting ecological changes in the region, agreeing with the arid characteristics of the upper reaches of the YRB [67].

Furthermore, this study focused on a restricted set of four indicators to evaluate EEQ in the Longyangxia to Lijiaxia Basin, highlighting the necessity for future research endeavors to incorporate additional parameters. These may include indicators such as gross domestic product (GDP), land use, and net primary productivity of vegetation (NPP) to ensure a more comprehensive and nuanced assessment of the EEQ in the study area. Numerous studies have been conducted on the time-series prediction of RSEI using machine learning and deep learning methods, yielding positive results [68,69,70]. However, this study did not consider predicting the future impact of spatiotemporal changes in EEQ. Future studies will employ advanced statistical models and methods such as GPs to address this issue [71,72].

## 5. Conclusions

This study used the GEE platform and Landsat TM/OLI remote sensing data to track the spatiotemporal dynamics and alterations in the EEQ within the Longyangxia to Lijiaxia Basin of the upper Yellow River over the past 31 years. This was achieved through the reconstruction of the RSEI. The results were as follows:

1. In the seven historical remotely sensed images acquired between 1991 and 2021, the average RSEI values ranged from 0.6 to 0.8 and showed decreasing, increasing, and decreasing trends. Notably, the largest percentage of areas with consistent EEQ over the 1991–2021 period indicates continued stable ecological conditions across much of the region.

2. Over the 1991–2021 period, the Moran’s I values for EEQ indicate a positive correlation in the spatial distribution of EEQ within the study area, and the spatial distribution is characterized by clustering rather than randomness. The H–H aggregation area initially decreased and subsequently increased, whereas the L–L clustering was predominantly situated in the basin along the coast and the western region. This indicates a clear change in aggregation characteristics over a specific time frame.

3. Between 1991 and 2021, the LST exhibited relatively substantial explanatory power, with a notable disparity compared with the other factors. In addition, the *q* value of the interaction between NDVI and WET was 0.937 in 2011, which had a greater impact on the spatial distribution of ecosystems in the region.

## Figures and Tables

**Table 2:** Eigenvalue and contributions of the four variables to PC1 in 1991, 1996, 2001, 2006, 2011, 2016, and 2021.

Indicators	1991PC1	1996PC1	2001PC1	2006PC1	2011PC1	2016PC1	2021PC1
NDVI	0.20	0.23	0.28	0.14	0.07	0.16	0.17
WET	0.82	0.83	0.79	0.72	0.70	0.75	0.70
LST	−0.40	−0.38	−0.35	−0.67	−0.59	−0.30	−0.29
NDSI	−0.38	−0.35	−0.42	−0.11	−0.39	−0.56	−0.63
Eigenvalue	1.35	1.37	1.50	1.03	1.14	1.42	1.32
Percent Eigenvalue	96.61	97.75	97.32	97.58	97.50	97.31	96.23

**Table 3:** Area and proportion of RSEI level in 1991, 1996, 2001, 2006, 2011, 2016, and 2021.

RSEI Level	1991		1996		2001		2006		2011		2016		2021	
	**Area** (${\mathrm{km}}^{2}$)	**Pct.** **(%)**	**Area** (${\mathrm{km}}^{2}$)	**Pct.** **(%)**	**Area** (${\mathrm{km}}^{2}$)	**Pct.** **(%)**	**Area** (${\mathrm{km}}^{2}$)	**Pct.** **(%)**	**Area** (${\mathrm{km}}^{2}$)	**Pct.** **(%)**	**Area** (${\mathrm{km}}^{2}$)	**Pct.** **(%)**	**Area** (${\mathrm{km}}^{2}$)	**Pct.** **(%)**
Poor	2289.34	6.55	1441.29	4.12	2038.98	5.84	1658.98	4.74	2297.30	6.57	2318.78	6.63	2080.60	5.95
Fair	4597.66	13.15	3022.62	8.65	4823.94	13.82	4185.67	11.97	5046.07	14.43	5557.97	15.90	5372.19	15.36
Moderate	10,152.87	29.04	7567.98	21.64	11,770.66	33.72	9255.09	26.47	10,171.56	29.09	11,876.11	33.97	11,620.11	33.23
Good	9881.58	28.26	10,938.81	31.29	11,029.07	31.59	12,203.72	35.19	10,601.81	30.32	10,515.81	30.08	11,020.34	31.52
Excellent	8043.62	23.00	11,994.38	34.30	5245.35	15.03	7561.62	21.63	6848.34	19.59	4694.97	13.43	4871.84	13.93
RSEI Mean	0.70		0.77		0.67		0.71		0.68		0.65		0.66	

**Table 4:** Change detection of RSEI level from 1991 to 2021.

Year		Deterioration Obvious		Deterioration Slight		Invariability	Improvement Slight		Improvement Obvious	
1991 to 2001	Change level	−4	−3	−2	−1	0	+1	+2	+3	+4
	Area/${\mathrm{km}}^{2}$	5.65	57.25	658.64	5700.52	23,944.75	3262.38	966.57	264.66	47.57
	Change area/${\mathrm{km}}^{2}$	62.90		6359.16			4228.96		312.23	
	Percentage/%	0.18		18.22		68.59	12.11		0.90	
2001 to 2011	Change level	−4	−3	−2	−1	0	+1	+2	+3	+4
	Area/${\mathrm{km}}^{2}$	43.75	280.43	1174.69	5026.37	20,677.35	5119.72	1979.39	525.15	81.15
	Change area/${\mathrm{km}}^{2}$	324.18		6201.06			7099.11		606.30	
	Percentage/%	0.93		17.77		59.23	20.34		1.73	
2011 to 2021	Change level	−4	−3	−2	−1	0	+1	+2	+3	+4
	Area/${\mathrm{km}}^{2}$	84.02	685.60	2270.09	5309.82	19,471.13	5582.36	1350.99	146.86	7.13
	Change area/${\mathrm{km}}^{2}$	769.62		7579.91			6933.35		153.99	
	Percentage/%	2.20		21.71		55.78	19.86		0.44	

**Table 5:** Factor detection of four indicators in 1991, 2001, 2011, and 2021.

RSEI Factors	1991			2001			2011			2021		
	***q***	***p***	**Sort**	***q***	***p***	**Sort**	***q***	***p***	**Sort**	***q***	***p***	**Sort**
NDVI	0.172	0	4	0.142	0	4	0.103	0	4	0.101	0	4
WET	0.448	0	2	0.251	0	2	0.218	0	3	0.196	0	2
NDSI	0.346	0	3	0.211	0	3	0.240	0	2	0.136	0	3
LST	0.745	0	1	0.392	0	1	0.513	0	1	0.469	0	1

## Data Availability

Data are contained within the article.
